# Interface of Multiple Sclerosis, Depression, Vascular Disease, and Mortality: A Population-Based Matched Cohort Study

**DOI:** 10.1212/WNL.0000000000012610

**Published:** 2021-09-28

**Authors:** Raffaele Palladino, Jeremy Chataway, Azeem Majeed, Ruth Ann Marrie

**Affiliations:** From the Department of Primary Care and Public Health (R.P., A.M.), Imperial College of London, UK; Department of Public Health (R.P.) and CIRMIS-Interdepartmental Center for Research in Healthcare Management and Innovation in Healthcare (R.P.), University “Federico II” of Naples, Italy; Queen Square Multiple Sclerosis Centre (J.C.), Department of Neuroinflammation, University College London Queen Square Institute of Neurology, Faculty of Brain Sciences, University College London; National Institute for Health Research (J.C.), University College London Hospitals, Biomedical Research Centre, London, UK; and Departments of Medicine (R.A.M.) and Community Health Sciences (R.A.M.), Max Rady College of Medicine, Rady Faculty of Health Sciences, University of Manitoba, Winnipeg, Canada.

## Abstract

**Background and Objectives:**

To assess whether the association among depression, vascular disease, and mortality differs in people with multiple sclerosis (MS) compared with age-, sex-, and general practice–matched controls.

**Methods:**

We conducted a population-based retrospective matched cohort study between January 1, 1987, and September 30, 2018, that included people with MS and matched controls without MS from England, stratified by depression status. We used time-varying Cox proportional hazard regression models to test the association among MS, depression, and time to incident vascular disease and mortality. Analyses were also stratified by sex.

**Results:**

We identified 12,251 people with MS and 72,572 matched controls. At baseline, 21% of people with MS and 9% of controls had depression. Compared with matched controls without depression, people with MS had an increased risk of incident vascular disease regardless of whether they had comorbid depression. The 10-year hazard of all-cause mortality was 1.75-fold greater in controls with depression (95% confidence interval [CI] 1.59–1.91), 3.88-fold greater in people with MS without depression (95% CI 3.66–4.10), and 5.43-fold greater in people with MS and depression (95% CI 4.88–5.96). Overall, the interaction between MS status and depression was synergistic, with 14% of the observed effect attributable to the interaction. Sex-stratified analyses confirmed differences in hazard ratios.

**Discussion:**

Depression is associated with increased risks of incident vascular disease and mortality in people with MS, and the effects of depression and MS on all-cause mortality are synergistic. Further studies should evaluate whether effectively treating depression is associated with a reduced risk of vascular disease and mortality.

Previous studies have demonstrated that depression is the most frequent comorbidity in people with multiple sclerosis (MS), affecting 21% to 24% of those with MS at any given time point.^1,2^ This is significantly higher than in the general population^3^ and is associated with multiple adverse outcomes, including disability progression and reduced health-related quality of life.^4,5^

In the general population, depression is associated with a 30% increased relative risk of developing vascular disease and a 70% increased relative risk of all-cause mortality.^6-8^ Moreover, the more severe the depression is, the higher the risk is of onset of vascular disease and associated mortality.^7^ However, much less is known about these associations in the MS population. A Danish study found that psychiatric comorbidity, including bipolar disorders and psychoses, was associated with increased all-cause mortality, but this this study did not include a control group and thus could not establish whether this association differed in magnitude from that in the general population.^9^ A Canadian study reported a greater than additive interaction between MS and depression on all cause-mortality.^10^ Possible sex-specific differences in the association among MS, psychiatric comorbidity, and vascular risk may also exist. Women with MS have a lower prevalence of cardiovascular disease^11^ but a higher prevalence of depression and higher risk of cardiovascular disease mortality.^12,13^

We aimed to assess whether the association among depression, vascular disease, and mortality differs in people with MS compared with age-, sex-, and general practice–matched controls. We also wanted to assess whether MS and depression produced greater than additive effects on vascular disease risk and mortality and to explore possible sex differences in these associations.

## Methods

### Study Design

We conducted a population-based retrospective matched cohort study over the period of January 1, 1987, to September 30, 2018, using data from the Clinical Research Datalink (CPRD) database, which included people with MS and matched controls without MS from England.

### Standard Protocol Approvals, Registrations, and Patient Consents

Ethics approval for the present study was obtained from the CPRD Independent Scientific Advisory Committee (protocol No. 18_279R).^14^

### Study Population

We conducted this analysis using a study population that has been described elsewhere.^12^ In brief, we selected people with MS on the basis of ≥3 diagnostic codes: prescriptions or events for MS as identified using diagnostic and management primary care codes (Read codes), ICD-10 codes, and prescription of disease-modifying therapies used solely for treating MS.^15^ When data linkage to secondary care data was possible, we also used Hospital Episode Statistics data to confirm the MS diagnosis (G35, ICD-10). Consistent with a previously adopted algorithm,^12^ after we selected those MS cases, we applied additional inclusion criteria, including (1) diagnosis after January 1, 1987, when MRI was more widely available to support the diagnosis of MS; (2) at least 1 year of continuous registration with the CPRD general practice before the first MS event to obtain information on key covariates at onset; (3) defined sex (female or male); (4) valid date of birth; (5) age ≥18 years at time of cohort entry; (6) MS events preceded the date of death; and (7) valid patient clinical records with respect to continuous follow-up and data recording defined by the CPRD definition of up to standard. The index date was defined by the date of the first MS diagnosis/event as the index date.

For each person with MS, we randomly selected 6 people without MS (controls) matched on age, sex, and general practice list. To be eligible for selection, controls' clinical data recorded during the study period had to be up to standard. To limit the likelihood of including any controls who might develop MS in the future, controls could not have any events for MS or any other demyelinating disease recorded (e.g., optic neuritis, transverse myelitis, acute disseminated encephalomyelitis, and CNS demyelination not elsewhere classifiable).^12^ To reduce the variance, we included multiple controls per case.^16^ The controls were assigned the index date of their matched MS case. All participants included in the study were followed up until the event of interest, the end of the study period (September 30, 2018), or their death, whichever came first.

### Study Variables

Consistent with prior research using CPRD data,^12,17-20^ we defined study variables using an amalgamation of comprehensive primary care codes and ICD-10 codes.^12,19^ Prescribing data were extracted with the use of British National Formulary codes.

### Outcomes

The following incident vascular diseases occurring after the index date were included as study outcomes: acute coronary syndrome, cerebrovascular disease, composite macrovascular disease (including acute coronary syndrome, cerebrovascular disease, and peripheral arterial disease), cardiovascular mortality, and all-cause mortality.

### Covariates

On the basis of their association with MS, depression, vascular disease, or mortality,^7,12^ covariates included age (continuous)**,** sex (female/male), ethnicity (White/non-White)**,** smoking status (nonsmoker/ex-smoker/current), type 2 diabetes (yes/no), and use of antihypertensive, antidiabetic, lipid-lowering, antiplatelet, and anticoagulant medications. We included the number of primary care visits in the year before the index year (continuous) to account for possible surveillance bias due to differences in health care use. We included the general practice Index of Multiple Deprivation (in quintiles)^21^ to account for socioeconomic status. We also included index year (categorical) as a covariate to account for temporal changes in care.

### Sensitivity Analysis

In the main analyses, we included use of medications as a proxy variable for clinical diagnoses to reduce the risk of misclassification. However, this may miss some affected individuals. Therefore, we conducted a sensitivity analysis in which medication variables were replaced by the following conditions: type 2 diabetes, hypertension, hyperlipidemia, and atrial fibrillation. These conditions were defined by the recording of both primary and secondary care diagnoses and medications, as appropriate (i.e., diagnosis of hypertension was based on primary and secondary care diagnoses of hypertension and on the use of antihypertensive medications).

### Statistical Analysis

In the index year, we stratified the study population into 4 groups: (1) controls without depression (reference group [R_00_]), (2) controls with depression (R_01_), (3) people with MS but without depression (R_10_), and (4) people with MS and depression (R_11_). We described the study population using means (SD), median (interquartile range), and frequency (percent). We used χ^2^, analysis of variance, and Kruskal-Wallis tests, as appropriate, to assess differences in baseline characteristics between groups. For study variables that showed significant results at univariate statistics, to compare differences between groups, we performed post hoc analyses using Bonferroni correction and Dunn tests, as appropriate. Differences between groups in the rates of incident vascular disease were assessed with Cox proportional hazard regression models. Individuals with conditions already present at baseline were excluded from the specific analysis (e.g., when we modeled differences in rates of acute coronary syndrome, we excluded those with a history of that condition at the index year from the analysis). Models were adjusted for covariates as described above. We included diagnoses of depression, diabetes, and vascular therapies as time-varying variables because of violations in the proportionality assumption. To assess the presence of a possible biological interaction between MS and depression on vascular disease and mortality using a departure from additivity effects,^22^ we estimated the relative excess risk of interaction (RERI) as R_11_ − R_01_ − R_10_ + 1 and the attributable proportion (AP) due to interaction as RERI/R_11_.^10,22-24^ Results were expressed as adjusted hazard ratios (HRs), RERI, AP, and 95% confidence interval (CI), as appropriate. For simplicity of presentation, given the inclusion of time-varying covariates in the regression models and that this is the metric most commonly used in vascular and mortality risk, we present HR at 10 years after the index date. All models were also stratified by sex. Results were considered statistically significant if *p* < 0.05.

We used Stata version 16 MP (StataCorp, LCC, College Station, TX) to conduct statistical analyses.

### Data Availability

The data that support the findings of this study are available from CPRD, but restrictions apply to the availability of these data, which were used under license from the UK Medicines and Healthcare products Regulatory Agency for the current study and thus are not publicly available.

## Results

Between January 1, 1987, and September 30, 2018, we identified 12,251 people with MS who met the inclusion criteria and 72,572 matched controls. In the index year, 21% of the people with MS (n = 2,535) and 9% of controls (n = 2,535) had a diagnosis of depression. In both cohorts, people with depression were more likely to be female and were younger than those without depression (Table 1). More than 40% of those with depression were also smokers in the MS and control groups. However, the vascular risk at index year was higher for those without depression; 7.7% of the people with MS without depression had diabetes at the index year, and 6% of them were taking antihypertensive medications, although the difference was not significant for the latter compared with controls without depression (5.7% for controls without depression; Table 1).

**Table 1:**
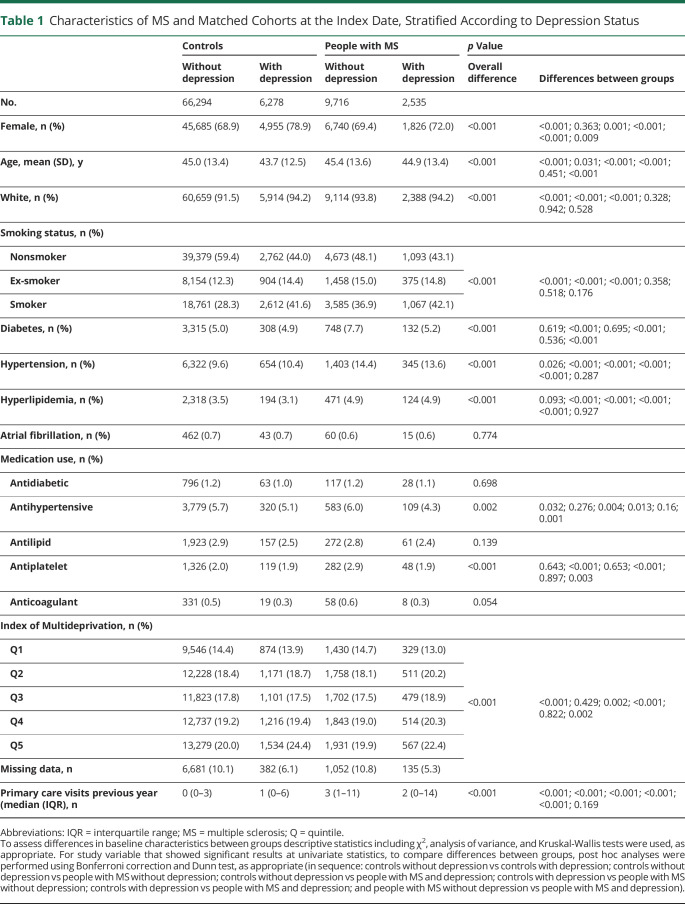
Characteristics of MS and Matched Cohorts at the Index Date, Stratified According to Depression Status

### Vascular Disease Outcomes

**Figure 1:**
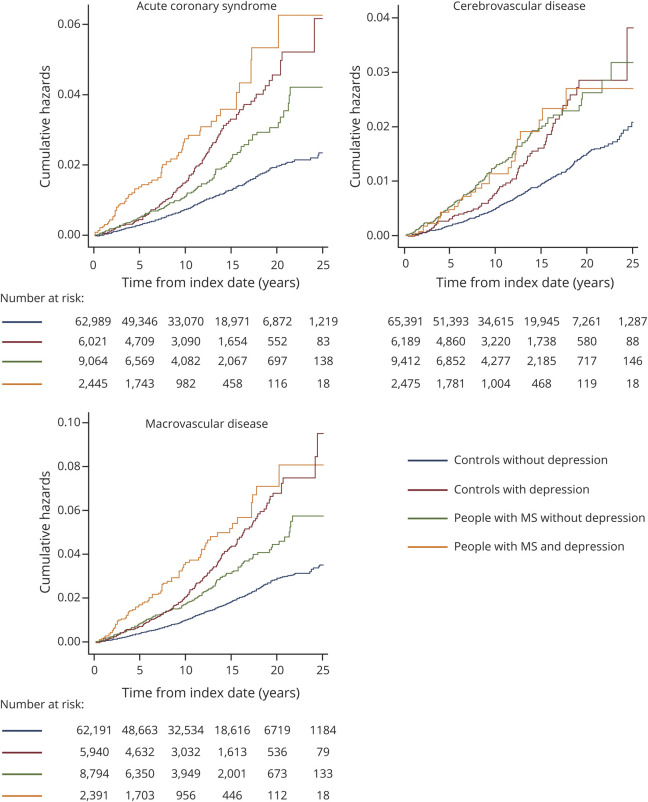
Cumulative Hazards of Macrovascular Events for People With MS and Matched Controls With and Without Depression For the definition of macrovascular disease, the following conditions were included: acute coronary syndrome, cerebrovascular disease, and peripheral arterial disease. MS = multiple sclerosis.

**Figure 2:**
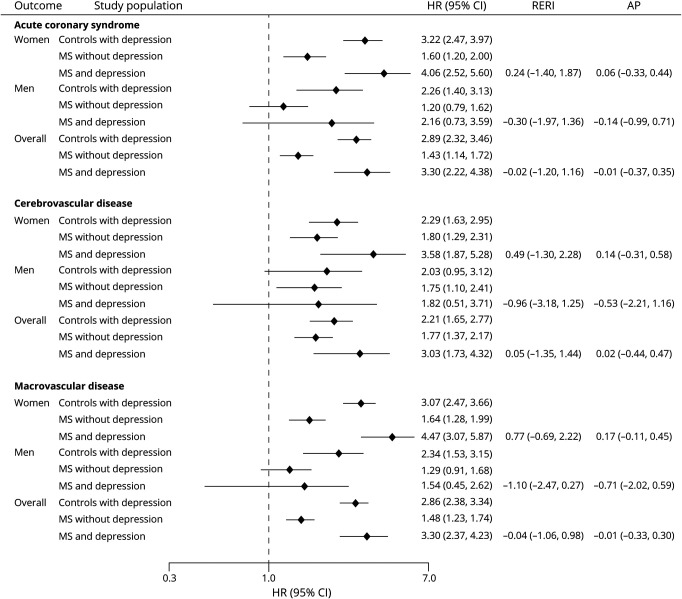
Association Among MS, Depression, and Risk of Macrovascular Disease Between January 1987 and September 2018 The association among multiple sclerosis (MS), depression, and vascular disease was assessed with Cox proportional hazard models adjusted for the following covariates: age (continuous); sex; ethnicity (White/non-White); smoking status (nonsmoker/ex-smoker/current); type 2 diabetes (yes/no); use of antihypertensive, antidiabetic, lipid-lowering, antiplatelet, and anticoagulant medications; number of primary care visits in the year before the index year; Index of Multiple Deprivation; and index year (categorical). Diagnoses of depression, diabetes, and vascular therapies were included as time-varying variables because of violations in the proportionality assumption. Results were presented as 10-year hazard ratios (HRs), relative excess risk of interaction (RERI), and the attributable proportion (AP) due to interaction. CI = confidence interval.

Over 10 years, the crude incidence per 100,000 person-years of any macrovascular disease was 0.66 (95% CI 0.60–0.72) for controls without depression, 1.34 (95% CI 1.08–1.65) for controls with depression, 1.17 (95% CI 0.97–1.42) for people with MS without depression, and 2.44 (95% CI 1.89–3.14) for people with MS with depression (Table 2). For each of the vascular outcomes of interest, including incident acute coronary syndrome, cerebrovascular disease, and macrovascular disease, the pattern of findings was similar (Figures 1 and 2). Compared to matched controls without depression, people with MS had increased risks of incident vascular disease regardless of whether they had comorbid depression. However, the risk was higher in people with MS and comorbid depression than in people with MS without comorbid depression (people with MS without depression: HR 1.48, 95% CI 1.23–1.74; people with MS and depression: HR 3.30, 95% CI 2.37–4.23). Controls with depression also had an elevated risk of incident vascular disease, similar to that of persons with MS and depression. We did not observe any departures from additivity for the association of MS and depression with incident vascular disease.

**Table 2:**
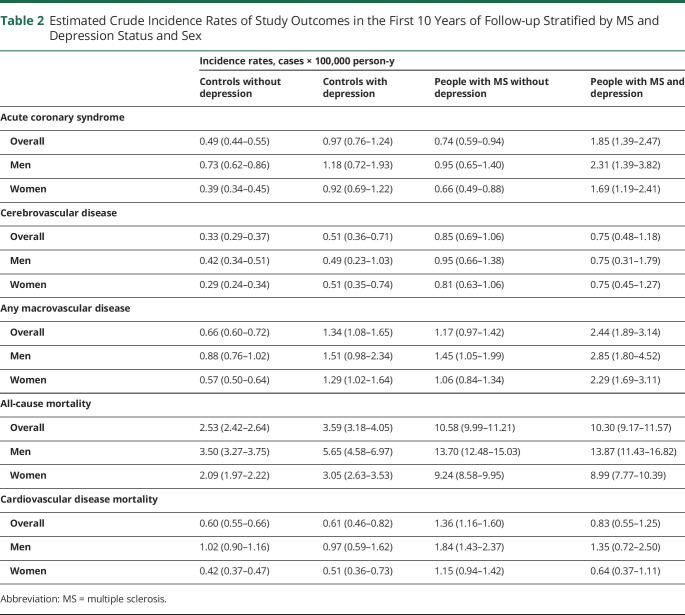
Estimated Crude Incidence Rates of Study Outcomes in the First 10 Years of Follow-up Stratified by MS and Depression Status and Sex

We found sex-related differences (Figure 2). Compared with women without depression, women with MS and depression had a greater risk of vascular disease than those with MS without depression. In contrast, among men, those with MS did not have statistically significant different hazards of acute coronary syndrome or composite macrovascular disease than controls without depression, although the direction of the effect was similar to what was observed in the population overall. With respect to cerebrovascular disease, men with MS who did not have depression had a 75% increased hazard of cerebrovascular disease (HR 1.75, 95% CI 1.10–2.41) over a 10-year follow-up period compared with controls without depression.

### Mortality Outcomes

**Figure 3:**
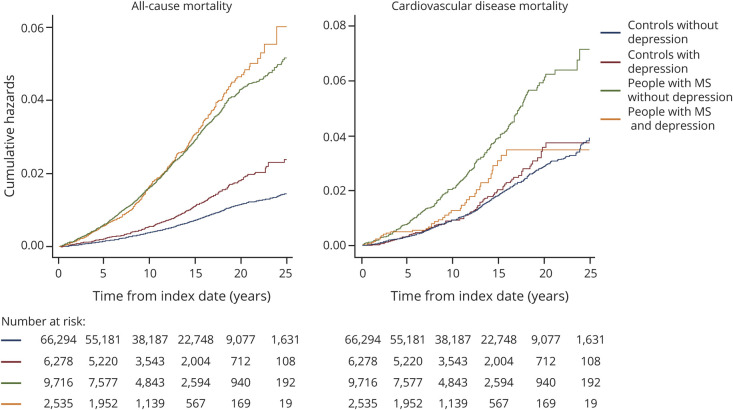
Cumulative Hazards of Mortality for People With MS and Matched Controls With and Without Depression MS = multiple sclerosis.

**Figure 4:**
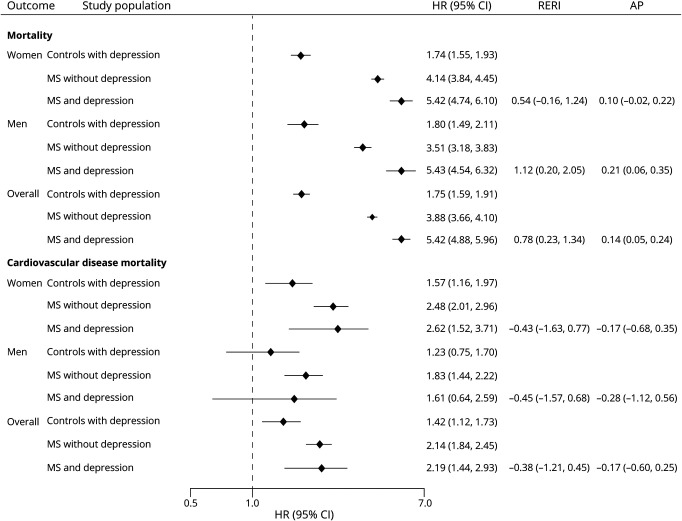
Association Between MS, Depression, and Mortality Disease Between January 1987 and September 2018 The association among multiple sclerosis (MS), depression, and mortality was assessed with Cox proportional hazard models adjusted for the following covariates: age (continuous); sex; ethnicity (White/non-White); smoking status (nonsmoker/ex-smoker/current); type 2 diabetes (yes/no); use of antihypertensive, antidiabetic, lipid-lowering, antiplatelet, and anticoagulant medications; number of primary care visits in the year before the index year; Index of Multiple Deprivation; and index year (categorical). Diagnoses of depression, diabetes, and vascular therapies were included as time-varying variables because of violations in the proportionality assumption. Results were presented as 10-year hazard ratios (HRs), relative excess risk of interaction (RERI), and the attributable proportion (AP) due to interaction. CI = confidence interval.

Depression was also associated with increased all-cause and cardiovascular mortality (Figures 3 and 4); the magnitude of the effect was greater for all-cause mortality. The incidence of all-cause mortality per 100,000 person-years was 2.53 (95% CI 2.42–2.64) in controls without depression, 3.59 (95% CI 3.18–4.05) in controls with depression, 10.58 (95% CI 9.99–11.21) in people with MS without depression, and 10.30 (95% CI 9.17–11.57) in people with MS and depression (Table 2). Compared with controls without depression, the 10-year hazard of all-cause mortality was 1.8-fold greater in controls with depression (HR 1.75, 95% CI 1.59–1.91), 3.9-fold greater in people with MS without depression (HR 3.88, 95% CI 3.66–4.10), and 5.4-fold greater in people with MS and depression (HR 5.43, 95% CI 4.88–5.96). The interaction between MS status and depression was synergistic, with 14% of the observed effect on mortality attributable to the interaction (overall: RERI 0.78, 95% CI 0.23–1.34; AP: 0.14, 95% CI 0.05–0.24). Analyses stratified by sex confirmed these findings. We also observed a synergistic effect for men (RERI 1.12, 95% CI 0.20–2.05; AP 0.21, 95% CI 0.06–0.35).

Depression was also associated with a 2-fold increased risk of cardiovascular disease mortality in persons with and without MS compared to controls without depression overall and in women. The associations were weaker in men; men with MS and depression did not have increased cardiovascular mortality. We did not observe any departures from additivity for the association of MS and depression with cardiovascular mortality.

### Sensitivity Analysis

Results from the sensitivity analysis including as covariates in the statistical models conditions identified according to diagnoses and medications rather than use of medications alone confirmed the findings from the main analysis for both incident vascular outcomes and mortality outcomes (Table 3).

**Table 3:**
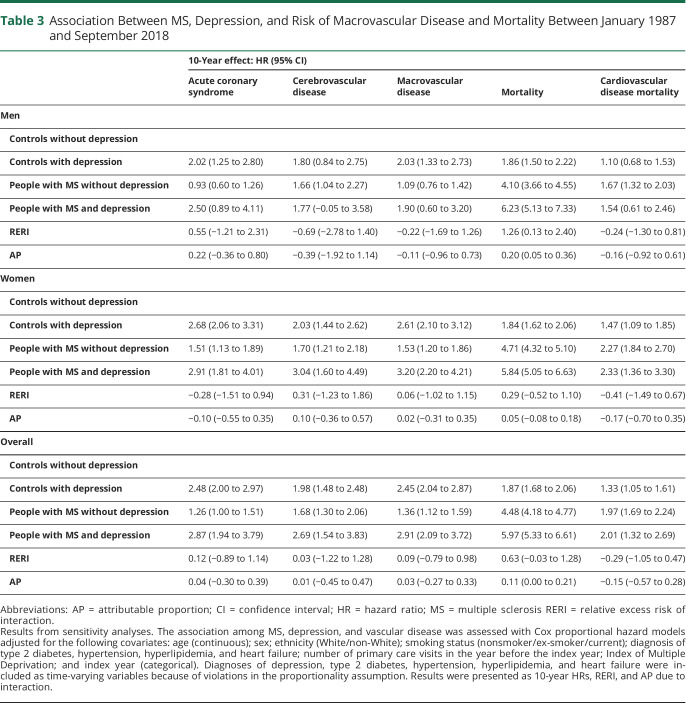
Association Between MS, Depression, and Risk of Macrovascular Disease and Mortality Between January 1987 and September 2018

## Discussion

We conducted a large population-based matched cohort study of 84,823 people with or without MS and with or without depression. We found that people with MS and depression had a greater risk of acute coronary syndrome, cerebrovascular disease, composite macrovascular disease, cardiovascular disease, and all-cause mortality than controls and people with MS without depression. For most outcomes, differences were more pronounced in women than in men. For all-cause mortality, 14% of the observed effect was attributable to the interaction between MS status and depression, rising to 21% when the analysis was restricted to men. We did not observe an interaction between MS status and depression on cardiovascular mortality.

We observed that MS is associated with increased risks of vascular disease that are not fully accounted for by traditional vascular risk factors such as diabetes, hypertension, and smoking.^12^ Depression is a nontraditional risk factor that appears to contribute to this risk. In the general population, depression is associated with increased risks of vascular disease and mortality.^7,25^ Depression is also associated with subclinical atherosclerosis, as measured by carotid intima-media thickness, even in young adults.^26^ To the best of our knowledge, prior studies have not examined the association between depression and subsequent onset of vascular disease in MS. However, prior studies have shown that comorbid ischemic disease is associated with an increased risk of incident depression in MS,^27^ as well as in other immune-mediated inflammatory disorders, including inflammatory bowel disease and rheumatoid arthritis. Among people with rheumatoid arthritis, higher levels of depressive symptoms are also associated with increased odds of subclinical atherosclerosis.^28^ Coupled with our findings, these prior studies suggest that bidirectional relationships exist between depression and vascular disease in MS.

A Danish study of 8,848 persons with MS found that a composite measure of psychiatric comorbidity that included bipolar disorder, schizophrenia, and other psychoses was associated with 2.4-fold increased risk of all-cause mortality.^9^ The role of depression was not assessed. Among 9,496 participants in the North American Research Committee on Multiple Sclerosis Registry, psychiatric comorbid conditions were also associated with an increased risk of mortality.^29^ A prior Canadian study of 5,496 persons with MS and 27,354 age-, sex-, and geography-matched persons without MS reported a synergistic interaction between MS and depression on all cause-mortality with an AP of 0.13 (95% CI 0.031, 0.23).^10^ This observation is highly consistent with our findings of a synergistic effect between MS and depression on all-cause mortality with an AP of 0.14 (95% CI 0.05, 0.24).

We observed sex-specific differences in our findings such that the association between MS and depression with an increased risk of acute coronary syndrome, cerebrovascular disease, and composite macrovascular disease was statistically significant in women but not in men. Given that the hazard ratios were similar and that the number of men with MS and depression (n = 710) was smaller than that of women with MS and depression (n = 1825), this should be interpreted cautiously. That said, sex-specific differences in vascular risk and management of vascular risk factors are recognized in the general population. For example, men and women differ with respect to their age-specific risks and presentations of vascular disease,^30^ and diabetes is a stronger risk factor for cardiovascular and cerebrovascular disease in women than in men.^31,32^ In analysis of data from 35,416 participants in the National Health and Nutrition Examination Survey, women were less likely to have controlled dyslipidemia, while men were less likely to have controlled hypertension.^33^

Several mechanisms may explain the association of depression with incident vascular disease and mortality. Depression is associated with inflammation, immune dysregulation, autonomic dysfunction including higher plasma levels of norepinephrine, and dysregulation of the hypothalamic-pituitary axis.^34-37^ These factors are thought to play a role in vascular risk and mortality but do not appear to fully explain the effects of depression on these outcomes.^31^ We can only speculate as to why synergistic effects of depression and MS were observed on all-cause mortality. This could possibly reflect adverse effects of depression on adherence to disease-modifying therapy^38^ or on depression-associated health behaviors such as smoking.^39^ It is also uncertain why synergistic effects of depression and MS on cardiovascular mortality were not observed. This may reflect lower statistical power due to fewer events and warrants further investigation as to whether causes of death differ across the studied groups.

Study strengths included the comprehensive assessment of the association among MS, depression, vascular risk, and mortality; population-based design; the large sample size that allowed us to conduct sex-stratified analyses; the assessment of possible additive interactions between MS and depression on the outcomes of interest; and the use of >30 years of follow-up data from primary and secondary care settings. Several caveats merit discussion. First, we included data from January 1987 to December 2018, a period during which changes in the standard of care for MS and vascular disease occurred. However, we adjusted for index year, and in a previous study using the same study population, we conducted a sensitivity analysis restricting data to only MS cases and matched controls with index year after the full implementation of the 2001 McDonald criteria in England, which showed results similar to those including the entire study period.^12^ Second, we favored using Cox proportional hazard regression models over competing-risk models to assess differences for cardiovascular disease mortality due to the relatively low event rates in the smaller groups and because we were interested mostly in directly quantifying the hazard ratios among those individuals who were actually at risk of developing the event of interest, as previously suggested.^40^ We presented results as 10-year hazard ratio because this is the most commonly used metric in vascular and mortality risk. Although the absolute estimates differ at other time points, the interpretation is unchanged (data not shown). Third, due to the presence of missing data, we could not include risk factors such as body mass index in our analyses. Although body mass index is an important vascular risk factor, its associated risk of macrovascular disease is relatively low.^41^ Not being able to account for this risk factor might have a greater impact on mortality, although this association is also modest.^42^ We were unable to evaluate the proportion of deaths due to suicide to see if this explained the excess mortality associated with depression in MS. However, the average annual suicide rate in the MS population, while elevated compared to a non-MS population, is relatively low (16.8 per 100,000 population).*^27^* Finally, when routinely collected data are used, miscoding, misclassification, and misdiagnosis may occur. However, the CPRD is a reliable, widely used data source and is subject to regular quality checks.^14^ Furthermore, we restricted MS diagnoses to people with ≥3 MS events recorded during the study period to improve the specificity of our case definition.

Depression is associated with increased risks of incident vascular disease and mortality in people with MS. The effects of depression and MS on all-cause mortality are synergistic. These findings build on our previous work that showed an overall 30% increased hazard of any macrovascular disease compared to a matched control population^12^ and underscore the importance of identifying depression in the MS population. Additional studies should be conducted to evaluate whether effectively treating depression in the MS population (perhaps with a lower threshold than those not affected by MS) reduces the risk of incident vascular disease and therefore reduces disability progression and mortality.

## Glossary

AP: attributable proportion
CI: confidence interval
CPRD: Clinical Research Datalink
ICD-10: *International Classification of Diseases, 10th revision*
MS: multiple sclerosis
RERI: relative excess risk of interaction
